# Genetic Characterization of a Listeria monocytogenes Serotype IVb Variant 1 Strain Isolated from Vegetal Matrix in Italy

**DOI:** 10.1128/MRA.00782-20

**Published:** 2020-08-13

**Authors:** Marina Torresi, Massimiliano Orsini, Vicdalia Acciari, Gabriella Centorotola, Valeria Di Lollo, Marco Di Domenico, Daniela Manila Bianchi, Maureen Wakwamba Ziba, Clara Tramuta, Cesare Cammà, Francesco Pomilio

**Affiliations:** a National Reference Laboratory for *Listeria monocytogenes*, Istituto Zooprofilattico Sperimentale dell’Abruzzo e del Molise G. Caporale, Teramo, Italy; b Istituto Zooprofilattico Sperimentale delle Venezie, Legnaro, Italy; c National Reference Centre for Whole Genome Sequencing of Microbial Pathogens: Database and Bioinformatic Analysis, Istituto Zooprofilattico Sperimentale dell’Abruzzo e del Molise G. Caporale, Teramo, Italy; d Istituto Zooprofilattico Sperimentale del Piemonte, Liguria e Valle d'Aosta, Torino, Italy; e Ministry of Fisheries and Livestock, Central Veterinary Research Institute, Lusaka, Zambia; University of Maryland School of Medicine

## Abstract

We report the chromosome and plasmid sequences of a strain of Listeria monocytogenes IVb variant 1, a recently emerging serotype, isolated in Italy from ready-to-eat vegetables.

## ANNOUNCEMENT

Listeria monocytogenes IVb variant 1 (lineage I), characterized by a 6.3-kb cassette typical of lineage II strains, is often associated with recurring outbreaks and sporadic cases (1). We report the whole-genome sequence of L. monocytogenes IVb variant 1 strain 2017-TE-6913-1-1, which was isolated from an official radicchio sample from a catering company in northern Italy in February 2017.

The strain was isolated according to the ISO 11290-1:2005 procedure (2), which is based on two enrichment steps on Fraser broth and selection on Ottaviani-Agosti agar medium; a single typical colony was then plated onto blood agar and incubated at 37°C for 24 h. DNA was extracted using a Maxwell 16 tissue DNA purification kit (Promega Italia Srl, Milan, Italy) according to the manufacturer's protocol. Species confirmation (3) and serogroup determination (4) returned the IVb variant 1 profile (5).

One nanogram of genomic DNA was used for library preparation with the Nextera XT DNA library preparation kit (Illumina, San Diego, CA) according to the manufacturer’s protocol. Sequencing was performed on the NextSeq 500 platform (Illumina) with the NextSeq 500/550 midoutput reagent cartridge v2 (300 cycles), which generated 2,666,952 read pairs (150 bp), corresponding to a theoretical coverage of 275×.

Quality control was performed with FastQC v0.52 (6), and trimming was conducted with a standalone command-line version of the FastQ positional and quality trimming tool available on the Orione platform (7), imposing a minimum quality score of 20 and a minimum length after trimming of 50 bp. The genome was *de novo* assembled using the SPAdes genome assembler v3.13 (8) with default parameters for the Illumina 2 × 150-bp sequencing strategy. A single pseudomolecule was generated by ordering scaffolds with abacas.pl v1.3 software (9), using the L. monocytogenes serogroup IVb strain F2365 genome sequence (GenBank accession number AE017262) as a guide.

Gaps were filled through several iteration cycles of GapFiller v1.10 (10) and Pilon v1.23 (11) software, which finally returned a complete genome sequence. Circularity was confirmed by treating the tail-to-head sequence as a gap, which was submitted to GapFiller v1.10 for filling. The final assembly, a single molecule of 2,953,624 bp (GC content, 37.9%), was reordered starting from the region potentially containing the *dnaA* gene. Among the contigs not included in the final sequence of the chromosome, only one (52,825 bp [GC content, 35.0%]) contained an origin of replication, as identified by PlasmidFinder v2.0.1 (12); it showed a significant match (99.98% similarity and 95% horizontal coverage) with an L. monocytogenes plasmid sequence isolated in the United States in 2015 (GenBank accession number CP044433), with similarity against the nonredundant database determined using BLASTn (13). The sequence was refined and annotated using Pilon v1.23 and Prokka v1.12 (14), respectively. Default parameters were used for all software unless otherwise specified. The remaining contigs, which returned matches against other *Listeria* plasmids but did not show any origin of replication, were discarded.

Both the chromosome and plasmid ascribable sequences were submitted to GenBank; for the former, PGAP annotation was requested (15) and returned 2,956 genes (2,858 protein-coding genes, 5 rRNA operons, 57 tRNAs, and 22 pseudogenes). Among them, the presence of the region containing the characteristic L. monocytogenes serogroup IVb variant 1 genes *lmo0737*, *ORF2110*, and *ORF2819* (16), corresponding to *HNT73_RS01375*, *HNT73_07170*, and *HNT73_07960* loci, respectively, was confirmed.

The plasmid sequence annotation returned 58 coding sequences, including genes conferring resistance to heavy metals (mainly copper and cadmium). In particular, the cadmium resistance genes were located between an IS*6* family transposase and an IS*NCY* transposase protein, thus suggesting their potential mobility.

### Data availability.

The whole-genome assembly of L. monocytogenes strain 2017-TE-6913-1 was deposited in GenBank under the accession number CP053357 and of the plasmid p2017-TE-6913-1 under the accession number MT459813. Raw reads have been deposited in the NCBI Sequence Read Archive (SRA) under the accession number SRR11922747. All of the records are linked to the BioProject number PRJNA261392.
